# Health-Related Characteristics of Older Adults Who Attend Congregate Meal Sites in the United States

**DOI:** 10.3390/geriatrics2030022

**Published:** 2017-07-14

**Authors:** Fatma G. Huffman, Joan A. Vaccaro, Edgar R. Vieira, Gustavo G. Zarini

**Affiliations:** 1 Department of Dietetics and Nutrition, Florida International University, Miami, FL 33199, USA; jvaccaro@fiu.edu (J.A.V.); gzarini@fiu.edu (G.G.Z.); 2 Department of Physical Therapy, Florida International University, Miami, FL 33199, USA; evieira@fiu.edu

**Keywords:** congregate meals, physical function, self-rated health, food insecurity, race/ethnicity, sex differences

## Abstract

The purpose of this study was to determine health-related characteristics of a representative sample of older adults who attend congregate meal sites in the United States, and compare races/ethnicities and sexes. Participants were adults, aged 60 years and older, (*N* = 901) of the *2015 Tenth Annual National Survey of Older American Act Participants (NSOAAP)*. Difficulties with mobility and Activities of Daily Living were common among older adults who attended congregate meals. Health-related characteristics differed by race/ethnicity and sex. Higher percentages of men reported eating more than half their calories from the site: 61.0% (53, 68), compared to women: 41.2% (33,50); while twice the number of women reported exercising at the site: 42.7% (36, 50), compared to 21.8% (16, 29) for men. Hispanics reported poor/fair health, food insecurity, diabetes, and poverty more often than White non-Hispanics. The odds of reporting that meals helped maintain independence were higher for persons with food insecurity: OR = 2.67 (1.05, 6.80) and those who reported eating half or more of their calories from the site: OR = 5.78 (2.36, 14.30). Strategies that consider preferences by sex and race/ethnicity are required at congregate meal sites to encourage mobility and healthy eating.

## 1. Introduction

Good nutrition is vital in maintaining the health and independence of vulnerable, older adults who are in social and economic need [1]. The Older Americans Act (OAA) requires that Nutrition Programs provide meals and related nutrition services that promote health and help manage chronic disease [2]. The initial OAA was enacted in 1965 by President Johnson, and was reestablished in 2016 as the Older Americans Act Reauthorization Act, S.192 by President Obama (National Committee to Preserve Social Security and Medicare [3]). Formally under the Agency of Aging, it is now part of the Administration for Community Living (ACL) within the Department of Health and Human Services (DHHS). OAA funding is for 56 state agencies, and is distributed to over 200 tribal organizations, two native Hawaiian organizations, more than 600 area agencies on aging and 20,000 local service providers [3]. Nutrition programs (congregate meals and/or home-delivered meals) are for adults 60 and older; however, they concentrate assistance to persons with the greatest social or economic need, such as low-income or older adults living in rural areas [3]. 

Congregate meals are required to provide one-third of the necessary dietary requirements per day in accordance with the Dietary Guidelines, and are served five days a week [4]. The type of food varies by location; however, it must be deemed safe and sanitary by meeting state and local foodservice codes. In addition, older adults provided feedback concerning the types of meals so that they are made appetizing to the demographic [4]. Thus, variety depends on local availability, budget, and food preferences. The purpose of OAA nutrition programs are to: (1) reduce hunger and food insecurity; (2) promote socialization; and (3) promote health and wellness [4]. Each state has flexibility with extra services, but all provide transportation, some form of nutrition education (web links, flyers, counselling), and either fitness services or information on neighboring older adult fitness programs [4]. The overall goal of the congregate meal program is to help older adults remain independent through nutrition, socialization, and exercise [4]. Of the sites that offer fitness classes, many contain an aerobic component geared toward improving balance, strength, and mobility. 

Mobility is essential to independent living, and diets high in fruits and vegetables provide antioxidants and vitamins, which are key nutritional determinants of mobility in older adults [5]. In addition, moderate physical activity reduces mobility impairment, and higher levels of physical activity are associated with lower mobility dysfunction in older adults [6]. However, older adults are not meeting nutritional guidelines on fruit and vegetable intake, and are not following physical activity recommendations, both of which are related to physical function, mobility and independence [7]. Less than one-third of older adults consume the recommended 3 or more servings of vegetables, and 31.4% report no physical activity in the past month [7]. 

The issue of poor nutrition and low levels of physical activity is likely to be even more prominent among older adults who attend congregate meal sites, due to their low socio-economic status and limited access to high nutritional value foods. Some findings indicated that older adults attending congregate meals are older, have poorer health, and are more-likely to have difficulties in activities of daily living than their counterparts, who do not go to congregate meal sites [1]. Despite the public health and clinical significance of government feeding programs, there is limited information concerning nutrition-related health characteristics of older adults who attend congregate meal sites in the United States [8]. Investigators reported lower than recommended intakes of all food groups, particularly dairy, and a decrease of 0.038 cups of vegetables per day for each year in age for Kansas congregate meal attendees, who were 80 years and older [9]. Assessing the health status and health characteristics of the vulnerable population who reported eating at congregate meal sites is necessary in order to guide the development of interventions tailored for community-dwelling, vulnerable, older adults. Therefore, the objectives of this study were to (1) determine health-related characteristics of a representative sample of older adults who attend congregate meal sites in the United States; and (2) assess differences among these characteristics by sex and race/ethnicity.

## 2. Materials and Methods 

### 2.1. Study Design, Ethics, and Population

This study is a cross-sectional analysis of data from the *2015 Tenth Annual National Survey of Older American Act Participants (NSOAAP)*. The data are available to the public, and were collected by the Administration for Community Living (ACL), US Department of Health and Human Services. The research protocol was approved by the Office of Management and Budget (OMB); the OMB Control Number for the 2015 Tenth NSOAAP is: 0985-0023. All participants signed an Informed Consent Form for public use of their data, and there are no identifiers in the public data set. A two-staged stratified selection of 312 out of 628 Area Agencies on Aging (AAoA) was conducted, and is described in detail at the Administration for Community Living website [10]. 

Briefly, the first stage was the selection of the AAoA, and the second stage was the selection of the services within the AAoA. Weighting of each service record was done separately. Weighting was done in four steps: calculation of base weights, nonresponse adjustment, trimming of extreme weights, and post-stratification adjustments to known population control totals [10]. Initially, base weights were computed by taking the inverse of the selection probability for each sampled client. Then the base weights were adjusted for nonresponse, followed by trimming of the extreme weights [10]. The base weight is the inverse of the overall selection probability of a client [10]. The base weight of a client can be obtained by calculating the base weight for an agency and multiplying that weight by the within-agency-level base weight of a client in a service within that agency [10]. Finally, a post-stratification adjustment was made using available control totals. Fay’s modified Balanced Repeated Replication (BRR) method was used for computation of the sampling variances of survey estimates. The nonresponse adjustment was done in two steps by performing separate adjustments for agency-level and client-level nonresponse [10].

The data for this study were extracted from Congregate Meals, which was one of the six services; other services included Home Delivered Meals, Homemaker Services, Transportation, the Family Caregiver Support Program, and Case Management. The 2015 NSOAAP Congregate Meals survey was conducted by telephone. Participants were asked about their socio-demographics, foods consumed at the site and throughout the day, degree of satisfaction with meals and services that were received, medical health, emotional health, functionality, and social life. Sample weights and variance estimation were applied to account for non-response. The study population included 901 adults ages 60 years and older who completed the 2015 NSOAAP Congregate Meals survey. 

### 2.2. Study Variables

The major independent variables were sex and race/ethnicity. The variable for race/ethnicity was constructed by coding the responses for Hispanics and subtracting Hispanics from other categories to create White non-Hispanics, Black non-Hispanics, Hispanics, and Others (including Asians, American Indians, and Pacific Islanders). Dietary intake at home and at the congregate meal site was collected for the survey. Specific questions about usual dietary consumption at home and at the site (for days attending OAA meals) were asked. Total servings of each food group (such as grain, fruit, vegetable, meat, bean, nut, and dairy) were calculated by trained researchers. After total servings per day were measured, participants were asked what proportion of all the food they eat in a day was consumed at the site (for days they eat at the site). Participants were asked whether, on days they attend OAA meals, if they usually ate each of the food groups. Difficulty in the activity of daily living was assessed by a series of questions, such as using the toilet, driving, light and heavy housework, preparing meals, keeping track of money. Based on responses, the interviewers created a binary category of difficulty with Activities of Daily Living (yes/no), with three or more considered affirmative. Dependent variables included key properties of diet (fruit and vegetable intake, proportion of calories from congregate meals, and perceived independence due to eating at the congregate meal site) and attributes of physical function (mobility, activities of daily living, number of medical conditions (collapsed to 0–3, 4–7, or 8+)). Whether or not a participant exercised at the congregate meal site was either affirmative or negative; however, exercise classes vary, and are not available at all facilities. Other health-related variables included the following: self-reported diabetes; education level; marital status; food security (binominal: having enough money to buy food/not enough money to buy food); poverty (<$20,000 per year); and self-rated health. Self-rated health was based on the question: “in the past 12 months, how would you rate your health?” Responses were collapsed from five categories (excellent, very good, good, fair or poor) to three categories: fair/poor, good, and very good/excellent). Self-rated health has been validated against actual health in older adults, and is an independent predictor of mortality [11,12,13].

### 2.3. Statistical Analysis

To determine the relationships among race/ethnicity and health factors, data were analyzed using SPSS version 24 with the module for complex analytical samples where *p* < 0.05 was considered significant. Sample weights were applied to account for the complex design (see methods). For more details, refer to the website [10]. The sample weights were provided in the data set and applied as a sampling plan in SPSS (IBM, Armonk, N.Y., USA). (Physical function, mobility, diabetes, and self-rated health by race/ethnicity and by sex were analyzed by cross-tabulations for complex samples using the Pearson’s Chi-square estimate. Logistic regression analysis for complex samples was used to assess characteristics associated with ‘reporting that congregate meals helped with independence’. An adjusted *F* variant of the second-order Rao Scott adjusted Chi-Square statistic was used to test significance. Model fit was considered with correct classification of cases at least 75% and a *p*-value of <0.01.

## 3. Results

Demographics of the study population are presented in Table 1 with weighted and unweighted frequencies. Most participants were between 65 and 84 years old, female, White non-Hispanic with high school or some college education, an income at or below the poverty level, single, and lived in either a rural or urban area.

The participants’ health indicators are presented in Table 2. There were similar proportions of participants across levels of self-rated health and with/without mobility impairments; 44% had difficulties with activities of daily living. Most participants reported having enough money to buy food. About one third of the participants had diabetes. Eighty percent thought the congregate meals helped them to eat healthier and two thirds thought they help them to be more independent. A higher proportion of the participants said they eat 3 or less servings of vegetable per day. The majority (95%) said they eat vegetables at the congregate meal site, but only 36% said they exercise at the site. Less than 40% of the participants did health screenings at the site and only 12% reported having received nutrition education though the program; yet, some form of nutrition education (pamphlets, web-links, or counselling) is made available through every OAA program.

Health-related factors presenting significant differences in proportions among races/ethnicities are presented in Table 3. Mobility impairments were most common among White non-Hispanics, but Hispanics reported poor or fair health at much higher proportion than the White non-Hispanics, and they also were the group with higher proportion of food insecurity, diabetes and poverty as compared to White non-Hispanics.

Health-related factors presenting significant differences in proportions between sexes are presented in Table 4. A higher proportion of males had more than half of their daily calories coming from the congregate meals, and considered that they ate heathier because of the congregate meals; whereas, a higher proportion of females exercised at the congregate meal sites and were poor.

Health-related factors presenting significant differences in proportions among self-rated health classifications (poor/fair, good, or very good/excellent) are presented in Table 5. A significantly lower proportion of the participants that reported having very good/excellent health considered that the congregate meals helped with their independence, had diabetes, considered that they were eating healthier because of the meals, were poor, and had difficulties in at least two activities of daily living. At the opposite end, a significantly higher proportion of the participants that reported having poor/fair health had diabetes and difficulties in at least two activities of daily living.

Health-related factors were compared to reporting that congregate meals helped maintain independence by complex logistic regression (Table 6). Odds of reporting that meals helped maintain independence were higher for persons with food insecurity (reporting fear of food running out) (OR = 2.67 (1.05, 6.80)) and those persons who reported eating half or more of their calories from the site (OR = 5.78 (2.36, 14.30)). Demographics, SRH, mobility, and reporting eating vegetables at the site were not significant for independence in the model.

## 4. Discussion

Mobility impairments and difficulties with activities of daily living were common among older adults going to congregate meal sites; almost half of the participants reported one of these issues. Exercise can reduce mobility impairments and improve function [6,14]. One third of the participants said they exercise at the sites (22% of men and 43% of women). A needs assessment for the types of exercise programs preferred by sex should be performed, since the literature is lacking this information [15], and fewer men participated in exercise programs at congregate meal sites compared to women. Molanorouzi, Khoo, and Morris [15] reported differences in the factors that motivate people to engage in physical activity, as well as in the preferred type of activity based on age and sex. Congregate meal sites need to consider people’s preferences when developing exercise programs. For example, an exercise program initiated in two congregate meal sites in Georgia recruited male and female volunteers for an aerobic program; 27 out of the 31 people who volunteered were female [16]. According to our results, exercise programs in congregate meal sites are being attended predominately by females. Exercise programs targeting males and considering their preferences are needed. 

In addition to physical activity, nutritional factors also affect older adult mobility [5]. However, only 12% of the participants said they had taken part in nutrition education to them at the site. Given the importance of proper nutrition for optimal health, availability of nutrition education at the sites and encouragement to participate are warranted. Nutrition education in congregate meal sites has been found to improve attendee’s knowledge of healthy eating [17]; however, interventions that successfully change eating behavior have not been reported to date. If nutrition education were available on a continuous basis at congregate meal sites with strategies to reach diverse attendees, longitudinal measures of fruit and vegetable consumption may be more promising. Community interventions to increase fruit and vegetable intake by older adults have been successful, particularly those that consider age-specific barriers in access or knowledge [18].

Congregate meal sites provide an opportunity for community-based, college internship programs with registered dietitians and physical therapy students, for example. Intern dietitians could provide ideas for low-cost, easy to prepare, healthy meals and intern physical therapy students could provide exercises to reduce mobility disability. Also, community-based agencies in South Florida were successful in training lay people and professionals to conduct evidence-based physical activity for seniors in churches, senior housing, senior centers, community centers, and parks [19]. The feasibility of implementing evidence-based physical activity programs at congregate meal sites has been established in the Midwest and in two Georgia counties [16,20]. However, dissemination and wide implementation still needs to be accomplished. The findings of our study further demonstrate the importance and urgency of implementing such programs.

There were significant differences between sexes and among races/ethnicities in the health-related characteristics of older adults going to congregate meal sites in the United States. These differences may need to be considered when developing meals, nutrition education, and exercise classes at the site. All racial/ethnic minorities 45–64 years old from a large population sample of California were significantly less likely to engage in vigorous physical activity compared to White non-Hispanics; however, this difference was no longer significant for older adults [21]. Instead, the investigators found that chronic disease was a motivating factor in participation in physical activity. Differences in food preference by race/ethnicity and sex were reported for older adults in nutrition programs [15]. These findings suggest that strategies to help engage participants receiving congregate meals to eat healthy and exercise at the site need to consider food preferences, and education should be aimed at lowering the risk of chronic diseases and their complications. Approaches to engaging older adults, particularly those who are frail, in exercise classes should consider that the social norms concerning vulnerability differ by sex [22].

Programs to improve mobility and activities of daily living, particularly for White non-Hispanics, are needed. Efforts to reach more older Hispanics and encourage them to attend congregate meal sites are particularly important, because this is a highly vulnerable population that reports poor/fair health at a much higher proportion than other groups, and has the highest proportion of food insecurity, diabetes and poverty. Poor/fair self-rated health was associated with low income, food insecurity, having difficulty with activities of daily living, having diabetes, and not participating in social activities. Higher education levels, not self-rated health, was associated with a higher proportion consuming the recommended five or more servings of fruits and vegetables.

The study has several limitations to note. Although this was a representative sample of persons attending congregate meal sites across the United States, the demographics are continuously changing. The study was cross-sectional, and causality cannot be determined. Health-related factors were assessed based on self-reporting; some behaviors, such as diet, could be biased by sex or race; however, the trained staff used techniques to assess portions of foods consumed at the site and at home. Participation in exercise was only considered at the site, and some participants may have attended fitness activities in other facilities. Moreover, some congregate meal sites may not have exercise facilities or classes. The sample was not big enough to determine relationships among races/ethnicities by sex. There was uneven distribution across variables, and the attendees were primarily White non-Hispanic females.

## 5. Conclusions

Mobility impairments and difficulties with activities of daily living were common among older adults going to congregate meal sites. Most older adults going to congregate meal sites in the United States did not meet the nutritional recommendations for fruit and vegetable consumption, and few reported receiving nutritional education and exercising at the sites. There were significant differences between sexes and among races/ethnicities in the health-related characteristics of older adults going to congregate meal sites in the United States. Women were twice as likely to participate in exercise classes at the sites as men. This may be due to gender differences in activity preferences. According to the findings of a focus group, older women considered holistic, group activities as important in healthy aging; whereas, older men stated competitive physical activity aids healthy aging [23]. These findings indicate the importance of need assessments to develop sex- and race/ethnicity-specific nutrition and physical activity strategies at congregate meal sites. Programs focused on improving mobility, particularly in White, non-Hispanics, and nutrition education targeting older Hispanics are particularly important, since Hispanics reported significantly higher fair to poor health than the other racial/ethnic groups, and had high proportions of food insecurity, diabetes and poverty compared to White non-Hispanics.

## Figures and Tables

**Table 1:** Demographics of the participants.

Variable	Category	Unweighted Frequencies	Weighted Frequencies	*p*
Age (years)	60–64	64	82,130	<0.001
	65–74	326	634,465	
	75–84	328	544,470	
	85 or above	183	294,371	
Gender	Male	284	525,537	<0.001
	Female	617	1,029,899	
Education	Less than high school	148	257,575	0.001
	High School or GED	311	506,764	
	Some college, business, vocational or technical training	278	508,567	
	College and above	162	369,719	
Income	Below $20,000	353	573,131	0.180
	Above $20,000	411	682,629	
Race	Black non-Hispanic	138	162,462	<0.001
	Other	31	49,713	
	Hispanic	44	200,749	
	White non-Hispanic	678	1,130,639	
Marital status	Currently married	353	610,145	0.004
	Not married	543	938,108	
Dwelling	Urban	391	890,879	<0.001
	Suburban	174	273,961	
	Rural	314	415,970	

*Note*: The weighted sample represents adults who attend congregate meals across the nation. The total weighed frequency equals 1,555,436, and the total unweighted sample size equals 901. Unweighted frequencies not adding to 901 respondents who answered either ‘not sure’ or who refused to answer. Race “Other” = Asian, American Indian, or Pacific Islander. Significance was considered at *p* < 0.05.

**Table 2:** Health-related characteristics of the participants.

Variable	Category	Unweighted Frequencies	Weighted Frequencies	*p*
Self-rated Health	Poor to Fair	249	459,489	0.337
	Good	316	498,687	
	Very good to excellent	332	593,637	
Food Security/Enough money to buy food	Yes	732	1,283,375	<0.001
	No	153	232,692	
Mobility impairment	Yes	476	753,353	0.690
	No	425	802,082	
Difficulty in activities of daily living	0	499	899,994	<0.001
	1	230	397,568	
	2+	165	250,148	
Number of medical conditions	0–3	233	473,039	<0.001
	4	125	223,459	
	5	133	175,832	
	6	118	217,063	
	7	103	173,735	
	8+	189	290,305	
Diabetes	Yes	280	489,893	<0.001
	No	618	1,037,741	
Congregate meals help eat healthier	Yes	706	1,205,829	<0.001
	No	180	331,904	
Congregate meals help be more independent	Yes	580	903,722	<0.001
	No	295	581,504	
Total servings vegetables/day	<4	366	676,639	0.006
	4	176	341,352	
	>4	322	499,288	
Eat vegetables at site	Yes	846	1,487,964	<0.001
	No	48	59,980	
Exercise at site	Yes	320	554,196	<0.001
	No	579	999,584	
Health screenings at site	Yes	344	567,871	0.001
	No	555	985,521	
Received nutrition education through the program *	Yes	104	180,955	<0.001
	No	793	1,369,511	

*Note: n* = 901, unweighted. Significance was considered at *p* < 0.05. * Nutrition education varies from counselling to information through web links or pamphlets. It is not possible to assess if the education was received at the site or through a program-related agency.

**Table 3:** Health-related characteristics with significant differences among races/ethnicities.

Parameter	Non-Hispanic Black	Other	Hispanic	Non-HispanicWhite	*p*
Mobility impairment	32.2 ^a,b^ (17, 53)	25.1 ^a^ (12, 46)	26.0 ^a,b^ (10, 54)	55.6 ^b^ (47, 64)	0.010
Poor or Fair self-rated health	35.1 ^a,b^ (18, 57)	19.7 ^a^ (8, 41)	69.3 ^b^ (46, 84)	22.2 ^a^ (16, 30)	<0.001
Food insecurity/Not enough money to buy food	30.0 ^a^ (21, 41)	15.4 ^a,b^ (6, 36)	39.5 ^a,b^ (11, 78)	9.1 ^b^ (7, 13)	0.018
Diabetes	44.0 ^a^ (27, 63)	43.0 ^a^ (21, 69)	58.7 ^a,b^ (40, 75)	25.8 ^a,c^ (22, 31)	0.001
Poverty (income < $20,000/year)	55.3 ^a,b^ (40, 69)	35.6 ^a^ (13, 67)	82.5 ^b^ (65, 93)	40.5 ^a^ (34, 48)	<0.001

*Note:* Data presented as percentage and 95% CI in parenthesis. Significance is based on the adjusted *F* and its degrees of freedom. The adjusted *F* is a variant of the second-order Rao-Scott adjusted Chi-Square statistic. “Other” includes: Asians, American Indians, and Pacific Islanders. Any columns that have the same letter are not significantly different from each other. For example, for mobility impairment the “Other” race/ethnicity had significantly lower percent of mobility impairment compared to White non-Hispanics. Hispanics had significantly higher poverty as compared to White non-Hispanics. Letters, a, b, c, show differences between the column groups of race/ethnicity. These groups are different when the letters differ. Significance was considered at *p* < 0.05.

**Table 4:** Significant differences in health-related characteristics by sex.

Parameter	Males	Females	*p*
>½ daily calories from congregate meal site	61.0 (53, 68)	41.2 (33, 50)	0.003
Exercise at the site (yes)	21.8 (16, 29)	42.7 (36, 50)	<0.001
Eating healthier because of congregate meals	83.8 (77, 89)	75.7 (70, 81)	0.044
Poverty (income < $20,000 per year)	34.9 (27, 44)	51.9 (45, 59)	0.001

*Note:* Data presented as percentage and 95% CI in brackets. Significance was considered at *p* < 0.05.

**Table 5:** Significant differences in health-related characteristics and self-rated health.

Parameter (Affirmative)	Poor/Fair	Good	Very Good/Excellent	Total	*p*
Meals help independence	73.1 ^a^ (60, 83)	61.4 ^a^ (54, 69)	50.1 ^b^ (45, 55)	60.8 (28, 36)	0.006
Diabetes	50.8 ^a^ (42, 60)	34.3 ^b^ (28, 41)	15.1 ^c^ (10, 23)	32.1 (28, 36)	<0.001
Eating healthier due to congregate meals	87.2 ^a^ (81, 92)	82.5 ^a^ (74, 89)	68.3 ^b^ (60, 75)	78.5 (74, 83)	0.001
Poverty (<$20,000/year)	58.9 ^a^ (47, 70)	47.5 ^a,b^ (41, 54)	34.6 ^b^ (27, 43)	45.5 (39, 52)	0.001
Food insecurity	27.0 ^a^ (14, 45)	12.9 ^a,b^ (8, 20)	7.9 ^b^ (5, 13)	15.2 (11, 22)	0.005
Difficulty in 2 or more activities of daily living	35.1 ^a^ (27, 44)	12.7 ^b^ (8, 19)	4.4 ^c^ (2, 8)	16.2 (12, 21)	<0.001
Socially active (vs. would like to do more)	36.6 ^a^ (21, 55)	60.0 ^a,b^ (51, 69)	71.8 ^b^ (61, 80)	57.7 (50, 66)	0.001

*Note:* Data presented as percentage and 95% CI in parentheses. Significance is based on the adjusted *F* and its degrees of freedom. The adjusted *F* is a variant of the second-order Rao-Scott adjusted Chi-Square statistic. Columns that have the same letter are not significantly different from each other. For example, a significantly higher percent of persons who reported very good to excellent health were more socially active compared to those who reported fair to poor health. Letters, a, and b show differences between the column groups of health. These groups are different when the letters differ. Significance was considered at *p* < 0.05.

**Table 6:** Health characteristics and reporting that congregate meals helped independence by complex logistic regression.

Variable	Parameter	OR (95% CI)	*p*
Food insecurity	Fear of running out of food	2.67 (1.05, 6.80)	0.040
	No fear of running out of food (reference)	1.00	-
Calories from congregate meals	Half or more	5.78 (2.36, 14.30)	0.002
	Less than half (reference)	1.00	-

*Notes*: Dependent variable: meals help independence (reference category = no). Model fit: corrected model, *p* < 0.001; 78.0% of the cases were classified correctly. The following variables in the model were not significant: race/ethnicity, *p* = 0.164; education, *p* = 0.540; self-rated health, *p* = 0.082; marital status, *p* = 0.127; age category, *p* = 0.054; mobile impairment, *p* = 0.137; and reported eating vegetables at the site, *p* = 0.221.
